# Role of HDACs in normal and malignant hematopoiesis

**DOI:** 10.1186/s12943-019-1127-7

**Published:** 2020-01-07

**Authors:** Pan Wang, Zi Wang, Jing Liu

**Affiliations:** 1 The Xiangya Hospital, Central South University, Changsha, 410005 Hunan China; 2 Molecular Biology Research Center and Hunan Province Key Laboratory of Basic and Applied Hematology, School of Life Sciences, Central South University, Changsha, 410078 Hunan China

**Keywords:** Histone deacetylases, Hematopoiesis, Hematological malignancy, HDAC inhibitor, Drug resistance

## Abstract

Normal hematopoiesis requires the accurate orchestration of lineage-specific patterns of gene expression at each stage of development, and epigenetic regulators play a vital role. Disordered epigenetic regulation has emerged as a key mechanism contributing to hematological malignancies. Histone deacetylases (HDACs) are a series of key transcriptional cofactors that regulate gene expression by deacetylation of lysine residues on histone and nonhistone proteins. In normal hematopoiesis, HDACs are widely involved in the development of various lineages. Their functions involve stemness maintenance, lineage commitment determination, cell differentiation and proliferation, etc. Deregulation of HDACs by abnormal expression or activity and oncogenic HDAC-containing transcriptional complexes are involved in hematological malignancies. Currently, HDAC family members are attractive targets for drug design, and a variety of HDAC-based combination strategies have been developed for the treatment of hematological malignancies. Drug resistance and limited therapeutic efficacy are key issues that hinder the clinical applications of HDAC inhibitors (HDACis). In this review, we summarize the current knowledge of how HDACs and HDAC-containing complexes function in normal hematopoiesis and highlight the etiology of HDACs in hematological malignancies. Moreover, the implication and drug resistance of HDACis are also discussed. This review presents an overview of the physiology and pathology of HDACs in the blood system.

## Introduction

**Fig. 1:**
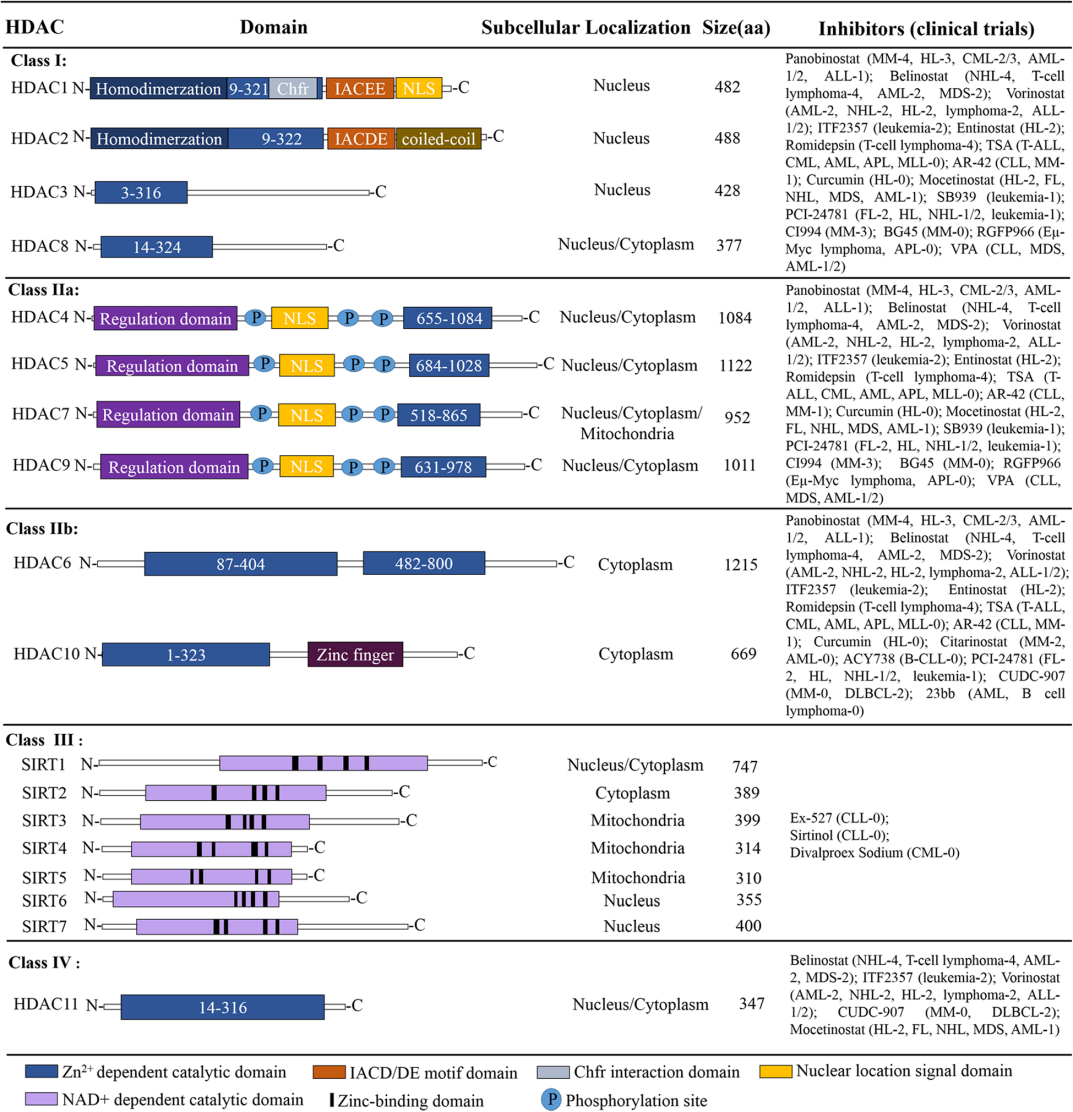
Classification of HDAC family

Epigenetic modifications play an indispensable role in the expression of hematopoietic lineage-specific genes. HDAC (histone deacetylase) and HAT (histone acetyltransferase) are two opposite classes of epigenetic modification enzymes. Generally, acetylation mediated by HATs opens the chromatin and allows gene transcription, whereas HDACs have a repressive effect on gene expression by deacetylating lysine residues on histone tails [1, 2]. Mammalian HDACs consist of 18 highly conserved genes [3], and they are divided into Class I (HDAC1, HDAC2, HDAC3, HDAC8), Class IIa (HDAC4, HDAC5, HDAC7, HDAC9), Class IIb (HDAC 6, HDAC 10), Class III (sirt1-sirt7) and Class IV (HDAC11) on the basis of phylogenetic analysis and sequence similarity to yeast factors (Fig. 1) [4]. Class I members are ubiquitously expressed with predominant nuclear localization [5, 6]. Furthermore, they are composed of approximately 400 amino acids and contain an N-terminal catalytic domain. Their catalytic domain is similar to a pocket and consists of two adjacent histidine residues, two aspartic acid residues and one tyrosine residue with Zn^2+^ ions as the core [7]. Class II members are more selectively expressed and can shuttle actively between the nucleus and cytoplasm [5, 6]. There are 600–1200 amino acids with an N-terminal regulatory domain that mediates the interaction with tissue-specific transcription factors and corepressors in class IIa HDACs. Importantly, there are two or three conserved serine residues in the class IIa N-terminal domain that can be phosphorylated by kinases, such as protein kinase D (PKD), which determines the nuclear export of class IIa HDACs [8, 9]. Class IIb members HDAC6 and HDAC10 contain another catalytic domain and an ubiquitin-binding zinc finger domain at the C-terminal region, respectively [7, 8]. The sirtuin family (SIRT1–7) of deacetylases represents class III, but they are functionally unrelated to HDACs; their deacetylase activity depends on NAD^+^ rather than on Zn^2+^-dependent enzymes [7]. As the smallest member of the HDAC family, HDAC11 is predominantly located in the nucleus, and more than 80% of the amino acid sequence is assigned to its catalytic domain [10].

As a series of epigenetic regulatory molecules, HDACs are involved in multiple aspects of hematopoiesis, such as stemness maintenance and lineage commitment. Abnormal expression or activity of HDACs and abnormal interactions between HDACs and transcription factors (TFs)/cofactors interfere with downstream gene regulatory networks, culminating in many different types of malignant hematopoietic diseases, such as lymphoma and leukemia [11]. Currently, HDAC inhibitors (HDACis) have been widely used as single agents or in combination with other chemotherapeutics in tumor treatment. However, therapy resistance remains a key problem. In this review, we focus on recent findings on the role of HDACs and their complexes in regulating gene expression during hematopoiesis or hematological malignancies, followed by a summary of combination therapy strategies and resistance mechanisms of hematological malignancies to HDACis.

## HDACs in hematopoiesis

**Fig. 2:**
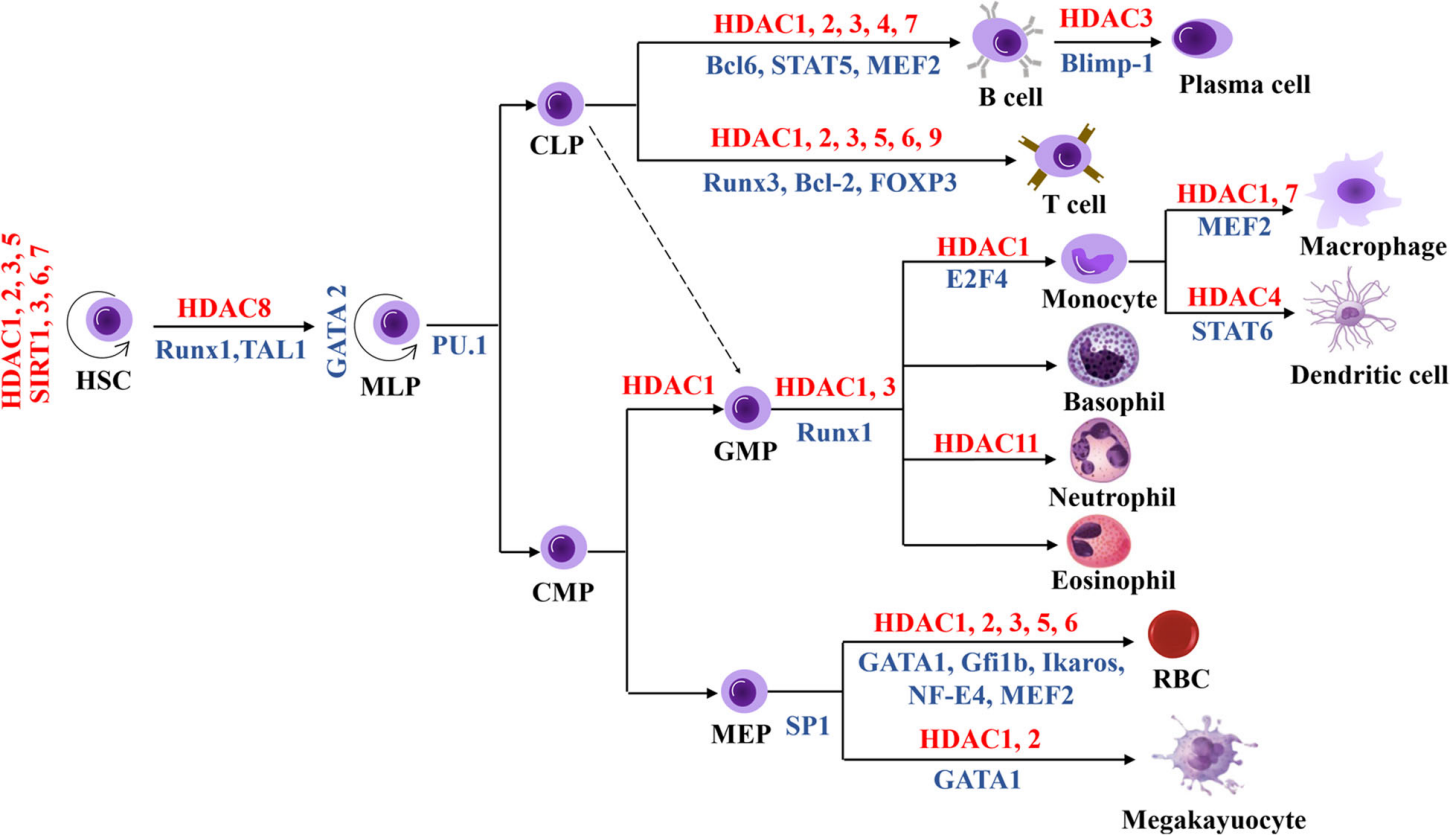
Schematic representation of the main HDACs and HDAC-related TFs involved in hematopoietic lineage commitment.

HDACs are extensively involved in multilineage development, including the hematopoietic stem cell (HSC)-progenitor lineage, granulocyte-monocyte lineage, erythropoietic lineage and lymphoid lineage (Fig. 2). During hematopoiesis, HDACs participate in the formation of a variety of transcriptional complexes where the reciprocal regulation between HDACs and TFs or other cofactors regulate histone acetylation levels, TF activity and functions of transcriptional complexes, which in turn modulate expression of various hematopoietic-related genes [12–17] (Table 1).

**Table 1:** HDAC-TF complexes in normal and malignant hematopoietic cells and their functions

TF(s)	Complex components	Cell type or disease	Function
GATA1	GATA1-acHDAC1	MEL cells	Promoting β-globin expression and erythroid commitment
	GATA1-HDAC3/HDAC4/HDAC5	COS cells	May repress GATA1 target genes and inhibit erythroid cell differentiation
	pERK-HDAC5-GATA1-EKLF	Human erythroblast	Inhibiting the transcription of globin genes
	GATA-1-Scl/TAL1-ET02-HDACs	G1E-ER-GATA1 cells	Participating in chromosomal translocation in AML
GATA2	GATA2-HDAC3/HDAC5	COS cells	Repressing the transcriptional activity of GATA2
GATA3	GATA3-Tbet-HDAC3/HDAC5	Jurkat cells	Regulating lymphocyte homing
EKLF	EKLF-HDAC1/Sin3A	K562 cells	Inhibiting β-globin gene expression
PU.1	PU.1-HDAC1/Sin3A	MEL cells	Inhibiting β-globin gene expression
	PU.1-Eto2- Sin3A-HDAC2	AML cells	Inhibiting myeloid differentiation genes, such as Mcsfr and Gmcsfr
Ikaros	Ikaros-GATA1-FOG1-HDAC1/NuRD	Mouse erythroid cells	Inhibiting γ-globin gene expression
Gfi	Gfi-1-G9a-HDAC1	HL60 cells	Repressing the expression of p21*Cip/WAF1*
	Gfi1b-CoREST-LSD1-HDAC 1/2	MEL cells	Committing hematopoietic differentiation
GFI1B	GFI1B-LSD1-RCOR1-HDAC1/2	Megakaryoblasts	Controlling megakaryoblast proliferation and differentiation
NF-E4	NF-E4 - HDAC1	K562 cells	Inhibiting γ-globin gene expression
E2F4	E2F4-RBL2-HDAC1-BRM (SWI/SNF)	Human monocytes	Repressing pluripotency stem cell factors in human monocytes
Runx1	Runx1-HDAC1/HDAC3	Human macrophages	Negatively regulating granulocyte formation
	Runx1-Eto2-Sin3A-HDAC2	AML cells	Inhibiting myeloid differentiation genes, such as Mcsfr and Gmcsfr
RUNX1/T1	RUNX1/RUNX1T1-HDACs-DNMTs	t(8;21) AML cells	Inducing leukemogenesis
	RUNX1/RUNX1T1-ETO-Sin3A-HDAC2	AML cells	Causing aberrant repression of late differentiation genes
MEF2	MEF2A/D-HDAC1/HDAC7	Human macrophages	Repressing the transcription of c-Jun
	MEF2-HDAC9	K562 cells	Activating γ-globin gene a and inducing HbF synthesis
	MEF2C-HDAC7	Human lymphoma	Silencing lineage-inappropriate genes in pro–B cells
TCF/LEF1	TCF/LEF1-SIRT6	Mouse stem cells	Inhibiting Wnt target genes and maintaining HSC homeostasis
Blimp-1	HDAC1/HDAC2	pre-B cells	Inhibiting c-myc transcription
STAT5	HDAC3-LSD1-STAT5	pro-B cells	Promoting the maturation of B cells
Bach2	Bach2-HDAC3-NCoR1/NCoR2-Rif1	Mature B cells	Increasing the deacetylation of Blimp-1 gene
BCL6	BCL6/SMRT-HDAC3	Human DLBCL cells	Establishing GC responses
	BCL6-HDAC4	Spleen B cells	Blocking B cell development and inducing uncontrolled cell proliferation
	BCL6-HDAC9	Mouse B-NHL cells	Deacetylating BCL6 and upregulating proliferation and survival genes
NF-κB	Sirt1-p300- NF-κB	Mouse T cells	Inhibiting transcription of Bclaf1
AML1	AML1-ETO-NCoR-mSin3-HDACs(1,2,3)	t(8;21) AML cells	Repressing AML1-mediated transactivation and activating leukemogenesis
	AML1-ETO-HDAC1/NCoR-SMRT	t(8;21) AML cells	Repressing AML1-mediated transactivation and activating leukemogenesis
	AML1-MTG16-HDAC1/3	t(16; 21) AML cells	Participating in nucleolar targeting
	TEL-AML1-HDACs	t(12;21) ALL cells	Repressing AML1 target genes
	AML1-MDS1-EVI1-CtBP1-HDAC1	MDS/CML/AM cells	Repressing gene transcription and inducing leukemia in mice
PML	PML-RARα-NCoR-Sin3-Ski/Sno-HDACs	t(15;17) APL cells	Inhibiting Rb and TRβ-mediated silencing and inducing leukemogenesis
PLZF	RARα-PLZF-HDAC1/NCoR-Sin3	t(11;17) APL cells	Impairing C/EBPα function and contributing to differentiation arrest in APL

### HSCs and progenitor lineage differentiation

HDAC1 and HDAC2 are essential regulators of HSC formation and homeostasis. Simultaneous deletion of HDAC1 and HDAC2 results in the loss of HSCs and, consequently, early hematopoietic progenitors, which are associated with the deregulated expression of genes linked to stem cell survival and maintenance, such as Dmkn, Nurcks1 and Tpt1 [18]. HDAC1 exhibits dynamic expression changes in cell lineage specification during hematopoiesis. Its expression is relatively low in human and mouse CD34+ hematopoietic progenitor cells (HPCs) and mature myeloid cells (monocytes and granulocytes). HDAC1 transcripts are moderately expressed in committed progenitors, erythroblasts and peripheral blood T lymphocytes. The unique expression pattern of HDAC1 is subject to regulation by hematopoietic transcription factors. For instance, HDAC1 transcription is repressed by GATA2 and C/EBP during common myeloid progenitor (CMP) differentiation into myeloid cells, especially granulocytes, and is activated by GATA1 and Sp1 during CMP differentiation into erythro-megakaryocytic cells [19, 20]. Specifically, knockdown of HDAC1 promotes hematopoietic progenitor differentiation toward the myeloid lineage with an increase in granulocyte/macrophage colonies and a reduction in the numbers and sizes of CFU-E and BFU-E, suggesting a role for HDAC1 in lineage commitment determination [6].

Class I HDAC3 was identified as a negative regulator in normal human HSC expansion. In vitro, HDAC3 silencing by HDACi-VA promoted CD34+ cell expansion without affecting differentiation potential [21]. Conditional deletion of HDAC3 in mice increased stem cells and early progenitor cells and blocked progress toward lymphoid-primed multipotential progenitor (LMPP) cells and lymphoid lineages, in which the loss of HDAC3 could impair S phase progression of multipotential progenitor (MPP) cells and, in turn, hinder cell DNA replication [22]. HDAC3 regulates the development of HSCs by interacting with HSC or HPC-specific TFs. For example, HDAC3 cooperates with Ncor2 to repress the fos-vegfd cascade by modulating the acetylation level in the fos promoter region, thereby mediating HSC formation [23]. HDAC3 directly interacts with GATA2 to inhibit GATA2–dependent targeting genes, which may be achieved by modifying the acetylation status of GATA2 in HPCs [24]. Another class I member HDAC8 is highly expressed in LT-HSCs, MPP and LMPP cells. HDAC8 plays a pivotal role in the maintenance and functional integrity of LT-HSC by deacetylating p53 [25].

Some class II and class III HDAC members are also involved in HSC homeostasis and aging. Class IIa HDAC5 has been shown to negatively regulate HSC homing by regulating p65 deacetylation [26]. Class III (SIRT1-SIRT7) members have been implicated in protecting HSCs from aging [27]^.^ Specifically, SIRT1 maintains aged HSC homeostasis by promoting nuclear localization and activation of FOXO3 and negatively regulating mTOR signaling [27–29]. Downregulation of SIRT3 in stem cells is associated with the aging of HSCs by regulating the global acetylation landscape of mitochondrial proteins and increasing ROS. While upregulation of SIRT3 can rescue functional defects in aged HSCs [30]. SIRT6 maintains HSC homeostasis by interacting with TCF/LEF1 and inhibiting the transcription of Wnt target genes via deacetylating H3K56ac [31]. SIRT7 maintains aged HSCs and prevents myeloid differentiation by repressing NRF1 (a key regulator of mitochondrial genes) and PFSmt (a mitochondrial protein folding stress factor) [32]. In addition, SIRT1 plays a critical role in promoting the mutation acquisition of CML, an age-dependent malignancy. Inhibition of SIRT1 sensitizes leukemic stem cells to imatinib treatment and blocks the acquisition of resistant BCR-ABL mutations by altering the function of DNA repair machineries in CML cells, and reduces the error-prone repair activity of DNA damage [33]. Further understanding of sirtuins in HSC aging and malignancy may provide novel treatment strategies to deter hematological aging and improve the treatment of hematological malignancies.

### Granulocyte-monocyte lineage terminal differentiation

**Fig. 3:**
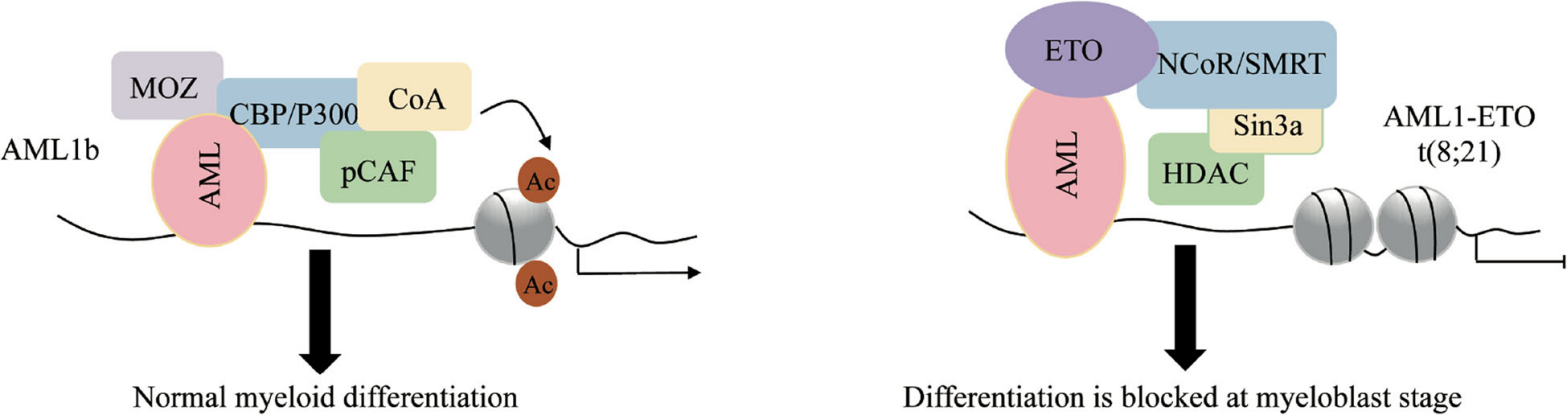
A model highlights component transformation in transcriptional complex is critical for leukemic tranformation.

In the bone marrow, granulocyte-monocyte terminal differentiation generally arises from upstream granulocyte-monocyte progenitors (GMPs), which have the potential to differentiate into granulocytes, dendritic cells (DCs) and monocytes/macrophages (Fig. 2) [34–36]. Class I and II HDACs are crucial for the proliferation and differentiation of bone marrow-derived monocytes (BMDMs) into macrophages and DC cells. For instance, under the treatment of TSA (a class I and II HDAC inhibitor), the amplification of murine myeloid progenitors is blocked and, in turn, differentiate into an elongated morphology of mixed M1/M2 profile, instead of the normal pancake-like shape of M1 inflammatory macrophages [37]. In addition, TFs associated with HDAC complexes shows dynamic changes during premonocyte to monocyte differentiation. For example, the differentiation-associated cell cycle exit induces E2F1 replacement with E2F4 at the PARP1 promoter and the assembly of an E2F4-RBL2-HDAC1-BRM (SWI/SNF) repressor complex which reduces PARP1 transcription and represses of pluripotent transcription factors such as POU5F1, SOX2, and NANOG [38]. Moreover, HDAC1 and HDAC7 have been implicated in macrophage terminal differentiation. Both of them interact with MEF2A/D heterodimers on the c-Jun promoter, thereby repressing the transcription of c-Jun, which is important for the development of the monocyte/macrophage lineage [39]. HDAC1 and HDAC3 are implicated in granulopoiesis. When Runx1 is phosphorylated by Src kinase, the reduced interaction of Runx1-HDAC1/HDAC3 is relevant to increased DNA affinity and the induction of granulopoiesis [40]. Class IIa HDAC4 can be recruited to the Arg1 promoter region, which leads to a reduction in the acetylation of both histone 3 and STAT6 proteins and subsequent transcriptional activation of Arg1, resulting in monocyte and CD8α(+) conventional DC differentiation [41]. Importantly, in some case, the aberrant recruitment of HDAC complexes by the oncofusion TFs to key hematopoietic genes is critical for leukemic transformation. For instance, in AML, AML1-ETO and RARalpha-PLZF fusions recruit NCoR/SMRT-HDAC1/3, while lose their binding ability with P300/MOZ/pCAF/CoA present at normal cells, thus reducing histone acetylation and producing repressive chromatin organization by HDACs, which results in gene transcriptional repression responsible for hematopoietic differentiation and, potentially, activation of a subset of other genes, including those for macrophage colony-stimulating factor and BCL-2 [42] (Fig. 3).

HDACs are able to regulate the transcription of granulocyte-macrophage colony-stimulating factor (MCSFR), granulocyte colony-stimulating factor (GMCSFR) and cytokines to control the development and activation of granulocyte-monocyte cells [34–36]. For instance, Runx1 and PU.1 independently interact with the ETO2-SIN3A-HDAC2 corepressor complex coactivate MCSFR and GMCSFR expression [43]. Additionally, class IV HDAC11 is gradually increased from promyelocytes to neutrophils differentiation. Knockout of HDAC11 in mice showed the expansion of maturing neutrophils and increases in TNF-α and IL-6 [2]. Importantly, several studies have shown that cytokines, including IL-2, IL-12, TNF-α and GM-CSF, regulate the innate and adaptive immune system and enhance immunity against FL, NHL and CLL [44]. The involvement of HDACs in cytokines transcription regulation in neutrophils propose a combination application of HDACis and cytokines, which potentially contributes to an enhanced tumor immuneresponse.

### Erythrocyte lineage terminal differentiation

**Fig. 4:**
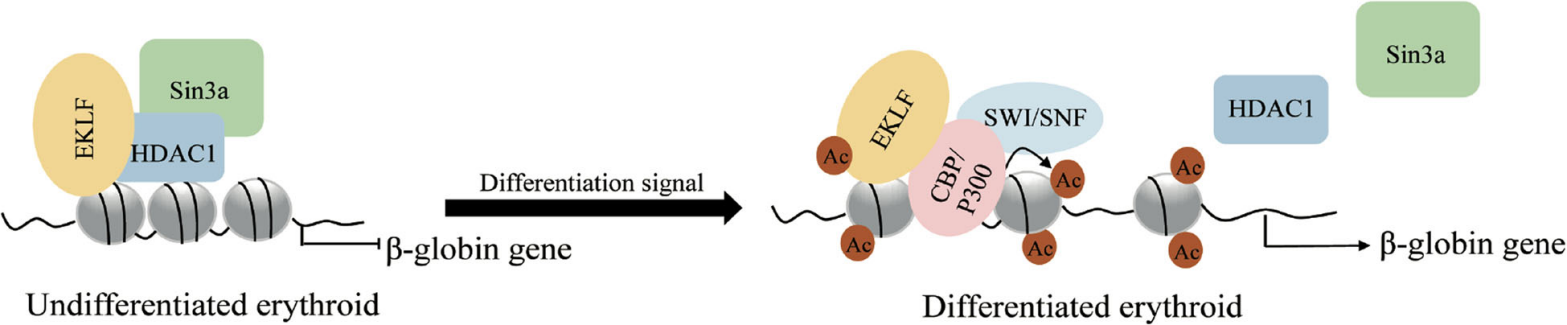
A model of CBP/P300 and HDAC component patterns determines the transcriptional function of TF in erythroleukemia cell differentiation.

Terminal erythroid differentiation begins with proerythroblasts, which subsequently undergo sequential mitoses, transforming into basophilic, polychromatic, and orthochromatic erythroblasts and enucleating into reticulocytes [45] (Fig. 2). The distinct interaction pattern between HDACs and erythroid-specific TFs plays an important regulatory role in erythropoiesis. HDAC1 and/or HDAC2 are the basic components of the SIN3A, NuRD and CoREST corepressor complexes [46, 47]. HDAC1/Sin3A can be recruited by EKLF to inhibit β-globin expression in undifferentiated EBHX11L cells, while this complex can be converted to the EKLF-p300/CBP-SWI/SNF complex and promote β-globin expression during the differentiation of EBHX11L cells to a primitive erythroid phenotype [48] (Fig. 4). Similarly, the PU.1-MeCP2-HDAC/mSIN3A complex inhibits the β-globin gene in undifferentiated MEL cells, and this complex is dissociated from the β-globin gene region during erythroid differentiation of MEL cells [49]. Interestingly, the acetylation state of the HDAC1/NURD complex plays a dual role during erythropoiesis. On the one hand, the HDAC1/NURD-GATA1 complex inhibits GATA2 expression by exerting deacetylation activity. On the other hand, p300-mediated acetylation of HDAC1 converts the NuRD complex from a repressor to an activator, which recruits GATA1 to promote β-globin expression and erythroid commitment [6, 50, 51]. Furthermore, the Gfi1b-LSD1-CoREST-HDAC1/2 complex is recruited to the c-myc promoter, leading to histone H3 hypoacetylation and c-myc transcriptional repression. C-myc repression is required for the arrest of the cell cycle and the initiation of erythroid differentiation [52].

Moreover, HDAC and erythroid-specific TF interactions are critical for the regulation of the γ-globin gene. For instance, the Ikaros-GATA1-FOG1-HDAC1/NuRD complex is required for silencing the human γ-gene during γ- to β-globin switching [53]. Diminished binding of acetylated NF-E4 to HDAC1 showed activation of γ-globin and inhibition of β-globin in fetal erythroid cells [54]. In addition, inhibition of HDAC3-NCoR (nuclear receptor corepressor) complex activity by the HDAC3-specific inhibitor SCFAD caused displacement of this complex from the γ-globin gene region with the recruitment of RNA polymerase II and upregulation of histones H3 and H4 acetylation status [55]. In contrast, HDAC9 might be recruited by MEF2 (myocyte enhancer factor 2) to the γ-globin gene promoter to mediate γ-globin activation and HbF synthesis during erythroid maturation of K562 cells [56]. Identification of HDACs-containing complex in association with globin gene switching may provide more molecular targets for intervening β-globin gene disorders.

**Fig. 5:**
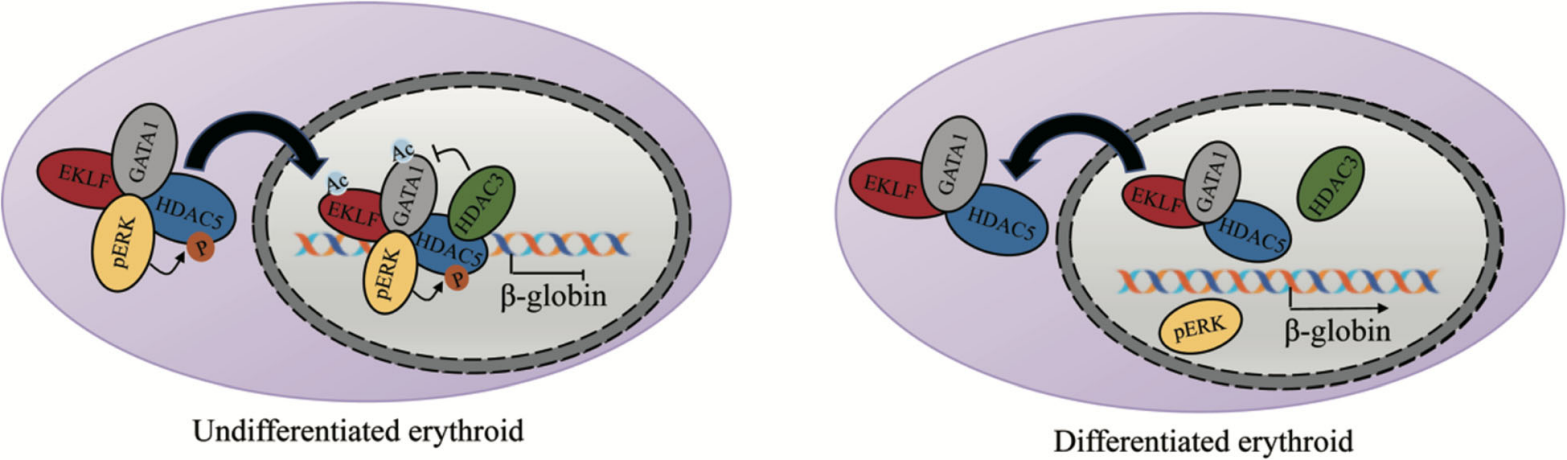
A model of class II HDAC interaction patterns in erythroid differentiation.

The function of class II HDACs that shuttle other proteins between the cytoplasm and nucleus is critical for erythropoiesis. For instance, Watamoto et al. found that although HDAC5 didn’t have deacetylation activity, it could shuttle GATA1 and EKLF from the cytoplasm to the nucleus via the formation of an erythroid-specific HDAC complex composed of HDAC5, GATA1, EKLF and ERK. Within the complex, the levels of p-ERK determine the shuttling activity of HDAC5. HDAC5 regulates the deacetylation levels of GATA1 and EKLF indirectly via the recruitment of HDAC3 to the complex [4, 5]. During erythroid maturation, HDAC5, GATA1 and EKLF remain associated, but the levels of pERK sharply decrease, which inhibits γ-globin expression [4] (Fig. 5). Furthermore, erythropoietin signaling induces the phosphorylation of HDAC5 via PKD, which promotes the dissociation of HDAC5 from GATA1 and GATA1 acetylation. Mice lacking HDAC5 showed resistance to anemic challenge, enhanced progenitor entry into the erythroid lineage and accelerated erythroid maturation in response to erythropoietin [9]. HDACs are also involved in the enucleation process of erythroid terminal differentiation. To date, HDAC6 and HDAC2 have been shown to play a role in chromatin condensation and enucleation. The former could promote enucleation via deacetylation of mDia2 (mammalian Diaphanous-related formin) and formation of the contractile actin ring (CAR). While HDAC2 specifically enhances enucleation but not differentiation or proliferation, where potential mechanism still needs to be further investigated [57, 58].

### Lymphocyte lineages terminal differentiation

The terminal differentiation of lymphoid lineages originates from common lymphoid progenitors (CLPs), which can be further divided into all-lymphoid progenitors (ALPs) and B cell-biased lymphoid progenitors (BLPs). ALPs retain the potential to generate B cells, T cells, natural killer cells and lymphoid dendritic cells, whereas BLPs are biased toward pre-B cells and immature B cells, and the latter migrate to the spleen to complete their maturation [59–61] (Fig. 2).

#### B cell

**Fig. 6:**
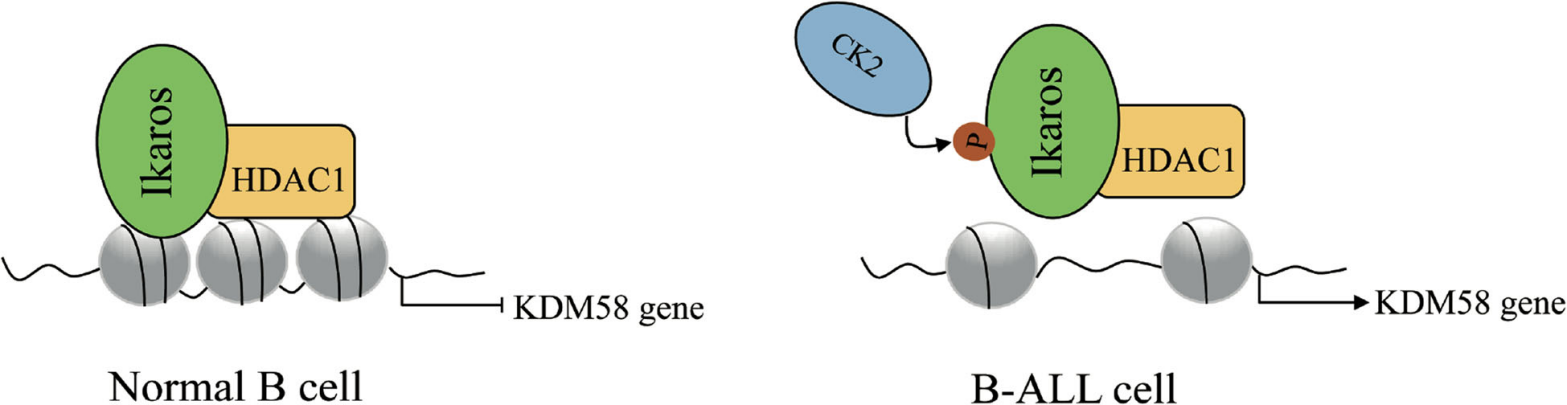
A model of TF modification affects the recruitment of HDAC to the promoter.

In early B cell progenitors, simultaneous deletion of HDAC1 and HDAC2 resulted in a dramatic block in B cell development at the pre–B cell stage by inducing p21 and p57 expression, accompanied by G1 arrest and apoptosis induction [62]. During terminal B cell development, Blimp-1 represses c-myc via recruiting HDAC1 and HDAC2. In mature B cells, although the loss of both HDAC1 and HDAC2 has no effect on viability, the cells fail to proliferate and undergo apoptosis [63, 64]. Similarly, in Eμ-myc-driven B cell lymphomas, the ablation of HDAC1 and HDAC2 prevents Eμ-myc tumorigenesis by decreasing proliferation and inducing apoptosis [65]. TFs have been found to mediate cross-talk between co-regulators in B cell development. For instance, the normal recruitment of HDAC1 by Ikaros is critical for the repression of the demethylase KDM58. In B-ALL, Casein kinase 2 (CK2)-mediated phosphorylation of Ikaros decreases HDAC1 recruitment to the KDM58 gene, which enhances KDM58 expression and leukogenesis [66, 67] (Fig. 6). For pregerminal center (GC) B cell differentiation, BCL6 RD2 domain-dependent recruitment of HDAC2 mediates repression of the trafficking receptors S1pr1 and Gpr183, contributing to the clustering of B cells within follicles [68]. Conditional knockdown of HDAC3 in early progenitor B cells of mice resulted in impaired B cell maturation and a defect in VDJ recombination [69]. Specifically, HDAC3 is implicated in different complexes at different B cell differentiation stages. For example, in pro-B cells, HDAC3 was identified as a component of the STAT5a-LSD1 complex, where it plays dual roles in determining the activation or repression of STAT5a [70]. In mature B cells, Bach2 recruits the HDAC3-NCoR1/NCoR2-Rif1 complex to repress Prdm1 transcription by deacetylating histone H3-K9, impeding the terminal differentiation of B cells into plasma cells [71].

HDAC-BCL6 complexes are implicated in the pathogenesis of lymphoma. For example, in normal GC B cells, CREBBP-regulated/active enhancers are counter regulated by the BCL6-SMRT-HDAC3 complex through a poised H3K27 deacetylation. However, in follicular lymphoma (FL) and diffuse large B cell lymphoma (DLBCL), CREBBP mutations disable its acetylation and result in unopposed deacetylation by the BCL6-SMRT-HDAC3 complex at enhancers of B cell signal transduction and immune response genes, thus promoting lymphomagenesis [72, 73]. In B cell non-Hodgkin lymphoma (B-NHL), aberrant expression of the HDAC9-BCL6 complex contributes to lymphomagenesis by altering pathways involved in proliferation and survival, as well as modulating BCL6 activity and p53 tumor suppressor function [74]. In contrast, as a corepressor partner of BCL6, HDAC4 plays an important role in suppressing leukemogenesis in complexes with BCL6 that recruit HDAC4 to repress oncogenes [75]. For example, in miR-155-induced pre-B cell leukemia/lymphoma, miR-155 directly targets HDAC4 and causes disruption of the HDAC4-BCL6 complex activity, resulting in derepression of BCL6 targets that block B cell development at an immature B cell stage and induce uncontrolled cell proliferation [76]. Hence, the interactions with different HDACs could confer different functional properties to TFs.

**Fig. 7:**
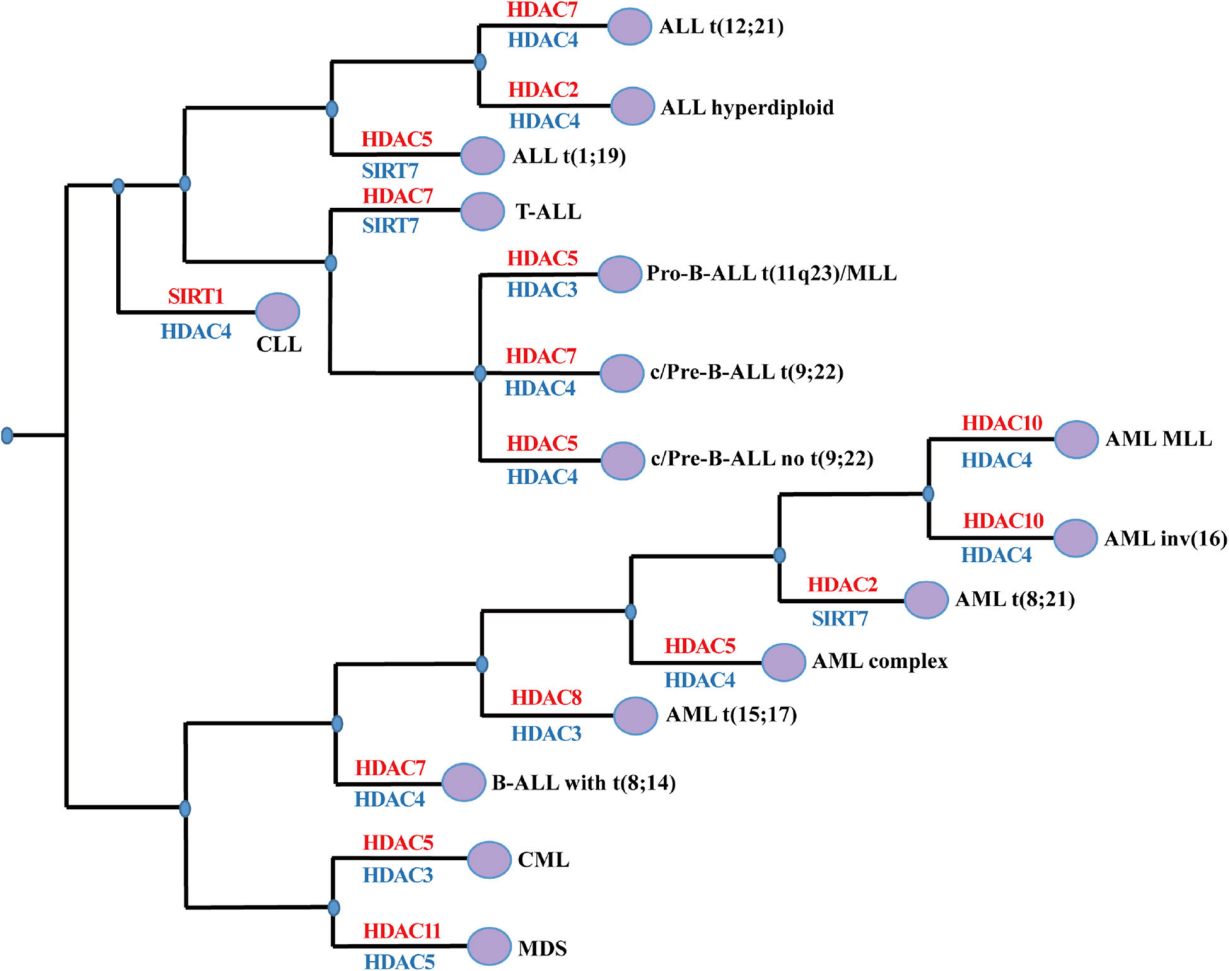
Abnormal gene expression of HDACS in different hematological malignancies.

Class IIa HDAC7 plays a physiological role in the lineage commitment of B cell progenitors. Conditional deletion of HDAC7 in mouse pro–B cells showed a block at the pro–B to pre–B cell transition, accompanied by severe lymphopenia in peripheral organs and pro-B cell lineage promiscuity. HDAC7 specifically interacts with the transcription factor MEF2C in pro-B cells, and the HDAC7-MEF2C complex is involved in silencing lineage-inappropriate genes, ensuring correct B cell differentiation. In particular, HDAC7 is frequently underexpressed in pro-B-ALL and Burkitt lymphoma. Ectopically expressed HDAC7 interacts with the MEF2C-HDAC3-SMRT complex and suppresses c-Myc expression in both MEF2C- and self-catalytic activity–dependent manners [61, 77, 78]. Conversely, HDAC7 appears to overexpressed in pre-B-ALL t (9;22), B-ALL t (8;14), ALL t (12;21) and T-ALL (Fig. 7), which suggest different expression patterns for HDACs in different hematological malignancies. In addition, HDAC6 is suggested to play a role in the regulation of immunogenicity in CLL and MM. The HDAC6 inhibitor ACY1215 or HDAC6 specific silencing resulted in the downregulation of PD-L1 in primary B cells isolated from CLL patients and restoration of CD4:CD8 ratio [79]. Similarly, the HDAC6 inhibitor ACY241 significantly reduces the frequency of CD138+ MM cells, CD4 + CD25 + FoxP3+ regulatory T cells, and decreases expression of PD1/PD-L1 on CD8+ T cells in bone marrow cells from myeloma patients [80]. More recently, the combination of HDAC6 inhibitor and anti-PD-L1 antibody can trigger cytotoxic T lymphocytes and NK cell-mediated MM cell killing. A combination of HDAC6 inhibitor, anti-PD1 and lenalidomide further enhanced the anti-MM immune response of MM cells induced by HDAC6 inhibition [81]. Although some HDACis such as Panobinostat has been shown to exert toxic effects on lymphocytes while several studies showed a stimulation of CD8+ T-cells activation and function upon pan-HDACi treatment [82]. These studies suggest that reasonable selection of HDACi is critical for immunotherapy in hematological malignancies.

#### T cell

During T cell development, HDAC1 and HDAC2 are essential for maintaining CD4 lineage integrity by inhibiting Runx3-CBFβ complexes that induce CD8 lineage programs in CD4+ T cells. Loss of HDAC1 and HDAC2 at the early stages of T cell differentiation result in a severe reduction in thymocyte numbers due to a block in cell cycle progression at the pre-TCR stage. Loss of these cells during late T cell development leads to reduced numbers of peripheral T cells and the appearance of CD4+ CD8+ T cells (TH) [83]. Similarly, Dovey et al. found that disruption of the HDAC1/2-Sin3A-NuRD complex by deletion of HDAC1 and HDAC2 in T cells of neonatal mice resulted in a marked reduction in thymocyte cellularity, a block in double-negative (DN) to double-positive (DP) transition, and a failure to proliferate in response to T cell receptor (TCR) signaling. Moreover, HDAC1/2 haploinsufficiency in mice causes a lethal pathology by T cell lymphomas with global histone acetylation and chromosomal instability [84], indicating an essential role for HDAC1/2 in the development of mature T cell populations and in maintaining genome stability.

HDAC3 is required for multiple stages in T cell development, including CD4 and CD8 lineage commitment, positive selection and peripheral T cell maturation. Specifically, HDAC3-deficient DP thymocytes fail to induce the CD4-lineage program and accelerate the redirection of MHC class II-restricted thymocytes to the CD8 lineage prior to positive selection with an increase in histone acetylation of CD8-lineage genes, such as Runx3 and Patz1 [85]. HDAC3 is required for positive selection of thymocytes. T cell development in CD2-icre HDAC3 conditional knockout (cKO) mice was blocked at positive selection due to a failure to downregulate RORγt (retinoic acid-related orphan receptor) and upregulate Bcl-2, which led to few CD4 and CD8 T cells. ChIP assays revealed that HDAC3 directly deacetylates histones to inhibit RORγt gene expression. The deletion of RORγt and transgenic expression of Bcl-xl corrects the positive selection defect in HDAC3-cKO mice [86]. HDAC3 is also required for T cell development from DN stage 4 into the early CD4/CD8 DP stage. The deletion of HDAC3 in the DN stage of thymocyte development by Lck-Cre-transgene caused a significant impairment at the CD8 immature single-positive (ISP) stage and the CD4/CD8 (DP) stage. When HDAC3^−/−^ mice were crossed with Bcl-xl-, Bcl2-, or TCRβ-expressing transgenic mice, CD4 and CD8 SP cells were partially rescued [87]. The NKAP-HDAC3 complex is required for post-thymic T cell maturation. The majority of HDAC3-deficient naïve T cells are recent thymic emigrants (RTEs), which cannot become long-lived naïve T cells. HDAC3-deficient peripheral T cells have a defect in TNF licensing after TCR/CD28 stimulation [88]. Moreover, the HDAC3-NCoR1/2-SMRT complex is essential for the normal development and suppressive functions of thymic and peripheral FOXP3^+^ T regulatory cells (Tregs) via association with FOXP3. The enzymatic activity of HDAC3 can be enhanced by NCoR1 or NCoR2/SMRT, which, in turn, deacetylates histone H3 at the IL-2 promoter and inhibits IL-2 transcription in FOXP3^+^ Tregs [89]. Therapy with pan HDACi such as TSA or SAHA can stimulate the thymic production of FOXP3+ Tregs and promote the peripheral conversion of murine and human T cells into Tregs [90].

Class IIa HDAC7 plays a role in life/death decisions in thymic T cell development. HDAC7 is exported from the nucleus by PKD during positive selection in thymocytes, and it regulates genes mediating the coupling between TCR engagement and downstream events that determine cell survival. Thymocytes lacking HDAC7 are inefficiently positively selected due to a severely shortened lifespan and exhibit a truncated repertoire of TCR Ja segments [91, 92]. Class IIa HDAC5 is implicated in Treg homeostasis. HDAC5^−/−^ mice showed reduced suppressive function and a decrease in Foxp3 in Tregs. CD4+ T cells lacking HDAC5 impair the ability of T effector cells to convert into induced Tregs. CD8+ T cells missing HDAC5 have a reduced ability to produce the cytokine of IFN-γ [93]. Class IV HDAC11 serves as a negative regulator of the T effector cell phenotype and function. T cells lacking HDAC11 show increased proliferation and proinflammatory cytokine production, such as IL-2 and IFN-γ, and inhibited tumor progression in murine lymphoma [94]. Specifically, HDAC11 and HDAC6 physically interact with each other and are simultaneously recruited to the IL-10 gene in antigen-presenting cells (APCs), where HDAC6 and HDAC11 act as a transcription activator and repressor of IL-10 expression, respectively [95]. Their dynamic interaction and the dynamic changes in the expression of IL-10 are suggested to explain the intrinsic plasticity of APCs in determining T cell activation versus T cell tolerance.

## The clinical implications of HDACis in malignant hematopoiesis

Aberrant expression of HDACs is linked to hematological malignancies, such as leukemias and lymphomas (Fig. 7). Specifically, the overexpression of HDAC5 and HDAC7 is associated with ALL, CML and AML. Low expression of HDAC4 is widespread in ALL, CML and AML [96–98]. To date, little is known about the mutation status and copy number alteration of HDACs in malignant hematopoiesis. However, by analyzing the TCGA database, we found that HDAC1, 4, 7 exhibited both gene mutations and CNAs in DLBCL, suggesting a key etiology for these HDACs (Table 2). However, their pathogenic mechanisms still need to be further investigated.

**Table 2:** HDACs with mutations or abnormal copy numbers in hematological malignancies

		Gene mutations			Copy number alteration (CNA)			
Disease	Gene	Mutation number	Case number with mutation	Percentage (total number)	Cytoband	Type of CNA	Case number with CNA	Percentage (total number)
AML	HDAC4	2	2	0.3% (622)	NA	NA	NA	NA
	HDAC7	NA	NA	NA	12q13.11	DEL (deletion)	1	0.5% (191)
CLL	HDAC4	1	1	0.2% (506)	NA	NA	NA	NA
DLBCL	HDAC1	1	1	0.7% (135)	1p35.2-p35.1	DEL (deletion)	1	2.1% (48)
	HDAC4	3	2	1.5% (135)	2q37.3	AMP (amplifications)	1	2.1% (48)
	HDAC7	1	1	0.7% (135)	12q13.11	AMP (amplifications)	2	4.2% (48)
						DEL (deletion)	1	2.1% (48)
MM	HDAC7	1	1	0.5% (205)	NA	NA	NA	NA
NHL	HDAC7	1	1	7.1% (14)	NA	NA	NA	NA

Notes: *NA* Not applicable; All data come from the TCGA database

Furthermore, HDACs are critical for the optimal oncogenic activity of leukemia fusion proteins. For example, AML1-ETO, PML-RARα and RARα-PLZF cause transcriptional repression of genes responsible for hematopoietic differentiation via recruitment of HDAC1/3, thus contributing substantially to leukemogenesis [99–107]. Given that the expression and activity of HDACs are closely related to the etiology of hematological malignancies, HDACs are hot targets for clinical drug development.

### The application of HDACis in malignant hematopoiesis

**Fig. 8:**
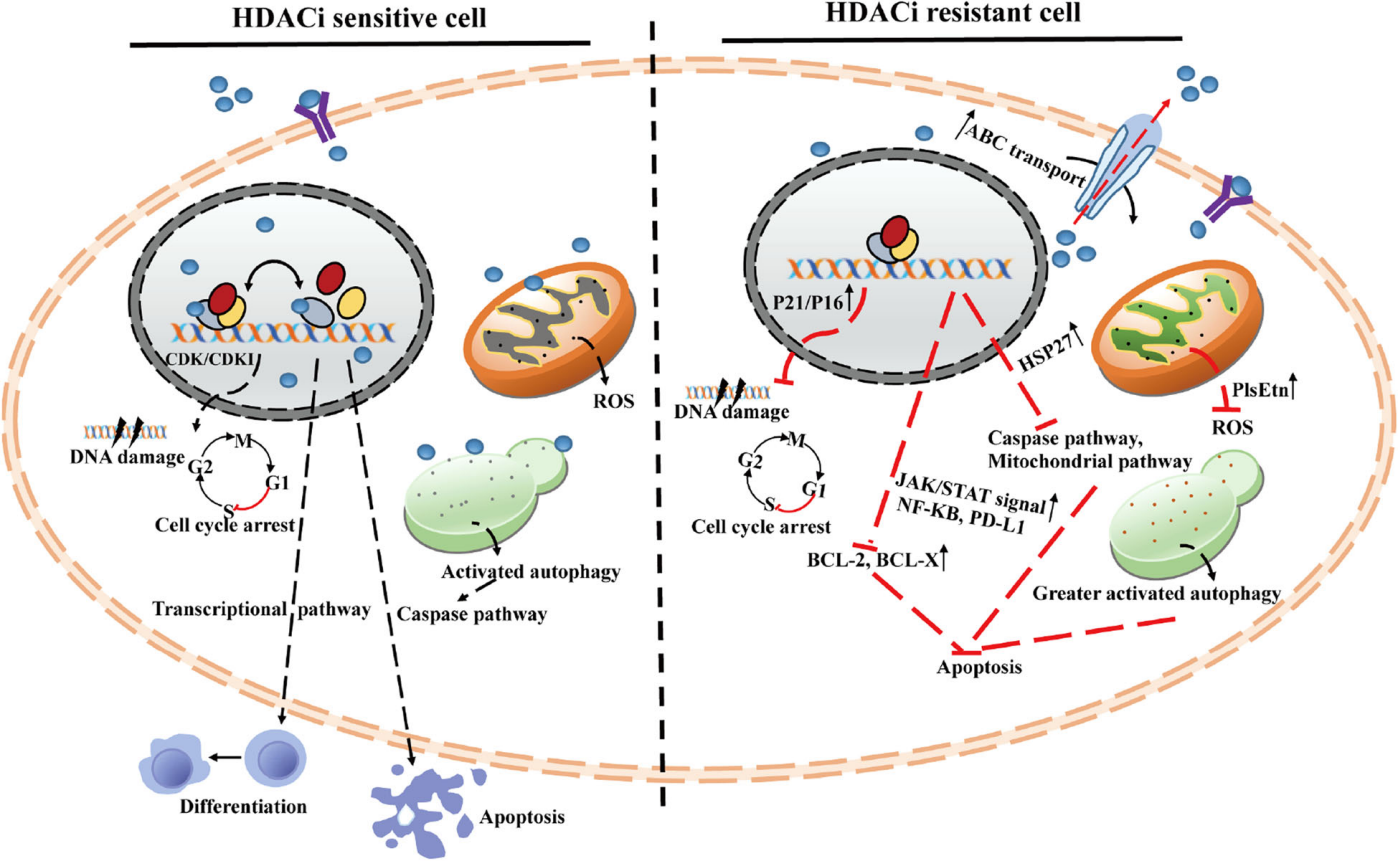
Sensitivity and resistance mechanisms of hematological malignancies to HDACis.

HDACis represent a class of cytostatic agents that interfere with the function of HDACs and are able to directly or indirectly regulate gene expression by inducing acetylation of histones or nonhistone proteins, involving cell-cycle arrest, promotion of differentiation or apoptosis and have different kinetics and activities depending on their chemical structures (Fig. 8). Generally, normal cells are often less sensitive to HDACis than tumor cells, and many HDAC inhibitors are undergoing extensive clinical evaluation as single agents and in combination with other chemotherapeutics [108, 109]. To date, panobinostat and belinostat have received FDA approval for the treatment of MM and NHL respectively. In addition, panobinostat, belinostat, romidepsin, entinostat and mocetinostat are in phase I, II or III clinical trials alone or in combination with other drugs for the treatment of other hematological malignancies (Fig. 1 and Table 3). Although, hydroxamate-based HDACis attract much attention in development of HDACi inhibitors, based on their remarkable zinc chelating capability. Nevertheless, it should be noted that some pan-HDACis, like romidepsin, panobinostat and vorinostat, display adverse effects, such as poor oral absorption, metabolic and pharmacokinetic problems because of glucuronidation, sulfation and enzymatic hydrolysis that lead to a short in vivo half-life [109]. Moreover, hydroxamate group can give rise to multiple off-target and mutagenic effects resulting from the coordination of other metalloenzymes, leading to undesirable adverse effects, such as nausea, thrombocytopenia, anemia and other metabolic issues, which may limit their clinical applications and promote the development of a new class of HDAC isoform-selective antagonists with reducing adverse effects [7, 110].

**Table 3:** HDACis in combination with other anticancer agents in phase I/II/III clinical trials

HDACis	Combination(s)	Cancer(s)	Clinical trial
Vorinostat	Sorafenib	AML, APL, MDS	I
	Carfilzomib	B-cell lymphoma	I
	Zolinza	Lymphoma or Leukemia	I/II
	Azacitidine	AML,MDS	II
	Temozolomide	AML	II
	Rituximab	Lymphoma	II
	Decitabine	AML, ALL, CLL, Lymphoma	I
		MDS	II
	Alisertib	Lymphoma	I
	Alvocidib	AML, CML, ALL	I
	Isotretinoin	APL,AML, Lymphoma	I
	Idarubicin	AML, CML, MDS	I
	Idarubicin, Cytarabine	AML, MDS	II
	Sorafenib, bortezomib	AML	I/II
	Cytarabine, Decitabine	AML, MDS	I
	AMG655, Bortezomib	Lymphoma	I
	Lenalidomide, Azacitidine	CML, MDS	II
	Lenalidomide, Dexamethasone	MM	I
	Bortezomib, Dexamethasone	MM	II
	Gemtuzumab, Ozogamicin, Azacitidine	AML	I/II
	Tacrolimus, Cyclosporine, Methotrexate	CML, AML, Lymphoma	II
Panobinostat	Carfilzomib	MM	I/II
	Bortezomib	T-cell lymphoma, MM	II, I
	Everolimus	Lymphoma	I/II
	Lenalidomide	HL, MM	II, I
	Placebo	HL	III
	Melphalan	MM	I/II
	Cytarabine	Leukemia, NHL	I
	5-Azacytidine	AML, MDS, CMML	I
	Decitabine	AML, MDS	I/II
	Everolimus	Lymphoma	I/II
	Imatinib mesylate	Leukemia	I
	Lenalidomide, Dexamethasone	MM	II
	Carfilzomib, Dexamethasone	MM	I
	Bortezomib, Dexamethasone	MM	II
	Bortezomib, Placebo	MM	III
	Dexamethasone, MLN9708	MM	II
	Dexamethasone, Lenalidomide, Bortezomib	MM	I
	Cytarabine, Daunorubicin	AML, MDS	I
	Ifosfamide, Mesna, Carboplatin, Etoposide, Pegfilgrastim	HL	I/II
Belinostat	Carfilzomib	Peripheral T-cell lymphoma, NHL, DCBCL, FL	I
	Rituximab	Lymphoma	II
	Idarubicin	AML	I/II
	Bortezomib	AML,ALL,MDS,CML	I
VPA	Decitabine	AML, MDS	II
	5-azacytidine	AML, MDS	II
	Rituximab, Cyclophosphamide, Doxorubicin, Vincristine, Prednisone	DLBCL	I/II
Romidepsin	Gemcitabine, dexamethasone and cisplatin	DCBCL	I
	5-azacitidine	Relapsed/refractory lymphoid maligancies	I/II
Mocetinostat	Brentuximab vedotin (SGN-35)	HL	I/II
	Azacitidine	MDS, AML	I/II
AR42	Decitabine	AML	I
	Pomalidomid	MM	I
Entinostat	Sorafenib tosylate	AML	I
4-PBA	Azacitidine	AML, MDS	I
SB939	Azacitidine	Hematologic Malignancies, MDS	I

Acquirement of the crystal structure of a given HDAC isoform combined with kinetic studies may contribute to overcome the structural homology between HDACs. For example, the availability of crystal structures of unique catalytic channels, such as catalytic domains CD1 and CD2 of HDAC6 and an acetate release channel of HDAC8, provided a unique strategy to develop their selective antagonists [111]. For the entrance ring area of catalytic channel, isoform selectivity could be achieved by designing zinc-binding groups bearing substituents that make specific interactions into the foot pocket (HDAC1–3) or into the lower pocket (class IIa HDACs) of a given isoform. For example, replacement of serine 107 by tyrosine in HDAC3 leads to a spatially restricted foot pocket that can be exploited to develop antagonists selective for class I HDACs [112]. Furthermore, the choice of surface-binding motifs that make specific interactions with the external characteristic grooves of the desired isoform, or targeting specific surfaces between HDACs and interacting partners that are critical for efficient deacetylase activity, such as the Ins (1,4,5,6) P4 binding site for HDAC1–3 or the CCHC zinc-binding motif for class IIa HDACs, might contribute to gain selectivity [113]. Finally, subtle structural differences in the hydrophobic active site channel have been exploited toward selective inhibitor design. Specifically, favorable interactions with a unique sub-pocket in the hydrophobic active site channel led to the creation of HDAC8-selective inhibitors [7, 114].

In addition, overcoming tumor heterogeneity and drug resistance promotes the development of combination treatment for HDACis. For example, a phase I study of vorinostat with decitabine-treated R/R AML patients who had mixed lineage leukemia (MLL) demonstrated a 35% composite complete response (CRC) rather than a 17% overall response rate (ORR) of vorinostat monotherapy [115]. Hence, combining HDACis with other chemotherapeutic agents is considered to be an effective way to enhance tumor drug sensitivity by improving the cellular efficacy and toxicity of HDACis to tumor cells [116–125] (Table 3 and Table 4). To date, the different mechanisms of HDACis combined with chemotherapeutic agents such as topoisomerase inhibitors, platinum-based chemotherapeutics, proteasome inhibitors, tyrosine kinase pathway inhibitors and epigenetic modifiers for advanced or drug-resistant hematological malignancies include (1) acetylating histones and inducing p21-CDK-mediated cell cycle arrest; (2) inducing apoptosis by regulating the expression of pro- and antiapoptotic genes through the intrinsic or extrinsic pathway; (3) inducing DNA damage and oxidative stress; (4) activating BTK (in CLL) or inhibiting ERK (in MM) and AKT (in CML) signaling pathways; and (5) regulating the expression of drug resistance-related molecules, such as downregulating BCR-ABL and upregulating Bim in hematological malignancies and downregulating CD44 in multiple myeloma (MM), NF-κB in ALL, γ-catenin in CML, and BRCA1, CHK1 and RAD51 in AML [126–145]. Specific combination strategies and their corresponding mechanisms are summarized in Table 3 [146–155]. Moreover, two-phase I clinical trials were carried out to assess the DNA methyltransferase (5-azacitidine) and HDACi (phenylbutyrate) for the treatment of hematological malignancies. A combination of BCL6 inhibitor (RI-BPI) with HDAC inhibitor (HDI) enhanced RI-BPI killing of primary human DLBCL cells in vitro [156, 157]. These studies suggest that the combination of HDACis with HDAC-interacting molecule inhibitors, such as TF inhibitors, chromatin remodeling molecule inhibitors or histone/DNA-modifying co-regulator inhibitors, is a potential combination strategies for hematological malignancies. However, optimizing combination scheduling and doses are necessary for avoiding pharmacological antagonism. For example, the combination of Vorinostat, Bortezomib and Pegylated liposomal doxorubicin (PLD) is suffering from the withdraw in phase I trials of MM. Since whole blood proteasome activity assays demonstrated a potential impact of Vorinostat on the chymotryptic-like activity of the proteasome [158].

**Table 4:** Mechanisms of HDACis combined with other agents in treating malignant hematopoiesis at preclinical settings

HDACis	Combination(s)	Cancer(s)	Mechanism(s)
Vorinostat (SAHA)	Bortezomib	Relapsed/refractory MM	Increasing p21 and cleaved PARP expression
		T-ALL	Inhibiting NF-κB signaling
	Carfilzomib or Bortezomib	Relapsed/refractory B cell lymphomas	Decreasing NF-κB activation and increasing Bim levels
	Rituximab	Lymphoma/leukemia	Increasing in p21 and acetylation of histone H3 leading to cell cycle arrest
	ABT-737	DLBCL	Inhibiting binding of BH3-only modulators and proapoptotic activators
	MG-132	Imatinib-resistant CML	Increasing intracellular ROS and repressing BCR-ABL expression
	S116836	Imatinib-resistant CML	Repressing antiapoptosis proteins Mcl-1 and XIAP, promoting Bim expression and mitochondrial damage
	BI2536	Imatinib-resistant CML	Triggering pronounced mitochondrial dysfunction, generating reactive oxygen species (ROS) and DNA damage
	KW-2449	Imatinib-resistant CML / AML	Inhibiting Bcr/Abl and inducing ROS and DNA damage
	ABT-737	Emu-myc lymphomas	Repressing BCR-ABL expression
	Idarubicin + Cytarabine	Advanced AML or Aza–resistant MDS	Generating reactive oxygen species (ROS)
Panobinostat (LBH589)	Bortezomib	Relapsed/refractory TCL	Increasing acetylation of HSP90, downregulating mitogen-activated protein kinase pathway signaling
	Carfilzomib	Relapsed/refractory MM	Inhibiting p97, HDAC or PI3Kα
	Ibrutinib	Relapsed/refractory MM	Generating ROS and inactivating ERK1/2
	ABT-199	Ibrutinib-resistant CLL	Reducing BTK/mutated BTK protein and signaling
	Ponatinib or Imatinib	AML	Upregulating Bim expression
	Everolimus	Imatinib-resistant CML	Forcing histone acetylation and decreasing BCR-ABL and AKT signaling
	Everolimus	HL/NHL	Activating the caspase pathway, inhibiting STAT5 and STAT6 phosphorylation, GLUT1 and mTOR
Romidepsin	Rituximab	Rituximab-resistant BL	Decreasing phosphorylated STAT3 binding to the MyD88 promotor
	ExPBNK	BL	Reducing p38 MAPK phosphorylation and enhancing MICA/B expression
	Ara-C	AML	Enriching Myc- and HOXA9-regulated gene pathways and inducing cell cycle arrest and DNA damage
	ATRA	APL	Inducing p21-mediated cell-cycle arrest and the expression of MDR1
	Gemcitabine, cisplatin and dexamethasone	DLBCL	Reducing LMP1 and c-myc expression
Belinostat	Vincristine or Paclitaxel	DLBCL	Inducing mitosis arrest and apoptosis
	Bortezomib	AML / ALL	Inhibiting NF-κB signaling and upregulating Bim expression
Entinostat	Sorafenib	Refractory/relapsed AML	Inhibiting HOXA9, MEIS1 and FLT3
	KW-2449	Imatinib-resistant CML / AML	Inhibiting Bcr/Abl, inducing ROS and DNA damage
Valproic acid	Decitabine	AML or MDS I/II	Inducing cell cycle arrest, DNA damage and apoptosis
	TRAIL/Apo2L	TRAIL/Apo2L-resistant CML	Increasing DR4 and DR5 expression
	ABT-737	Emu-myc lymphomas	Restricting Bcl-2 and Bcl-X_L_
	Chloroquine (CQ)	AML	Inducing *RASSF1A* expression and inhibiting autophagy
MGCD0103	Cytarabine or daunorubicin	AML	Inducing DNA damage and apoptosis
	Brentuximab vedotin	Relapsed/refractory HL	N/A
	Azacitidine	High-risk MDS or AML	Increasing p15 and caspase-3 expression
AR-42	Decitabine	M5 subtype-AML	Elevating miR-199b expression
	Lenalidomide	Lenalidomide-resistant MM	Upregulating miR-9-5p, downregulating IGF2BP3 and CD44
Depsipeptide	ATRA	APL	Upregulating of MDR1 and inducing p21-mediated cell cycle arrest
SBHA	ABT-737	Relapsed/refractory MM	Upregulating Bim expression and disabling cytoprotective autophagy
JSL-1	Imatinib	Imatinib-resistant CML	Inhibiting γ-catenin
Sodium phenylbutyrate	Azacitidine	AML or MDS	Reducing endoplasmic reticulum (ER) stress and ablating CHOP protein

Notes: *NA* Not applicable

NIH clinical trial database: www.clinicaltrials.gov. (These trials have been completed or are in active).

### Drug resistance mechanisms

Although HDACis play a tremendous role in improving patient survival and symptom control, in most cases, hematological malignancy cells develop drug resistance to HDACis, resulting in malignant phenotype regeneration and maintenance. Resistant-related proteins and abnormal in epigenetic or genetic factors and pathways are implicated in resistance to HDACis, including drug efflux, target status, chromatin alteration, upregulation of oxidative stress response mechanism, defects in proapoptotic pathways, and upregulation of antiapoptotic signals/stimuli (Fig. 8). For instance, SAHA induced multidrug resistance-related ABC transporter genes (MDR1, BCRP, MRP7, and MRP8) in leukemia cells. Overexpression of these cellular pumps has side effects on broad-spectrum drug resistance and cell intake. Changing the permeability proprieties of HDACis, adjusting the sequence of treatment or adopt nano-packaging materials may improve the efficacy of HDACis [159]. Furthermore, HSP72, as the most overexpressed protein in CTCL cell lines, induces chemoresistance against SAHA and VPA by suppressing the activation of caspase-3/8/9 and the mitochondrial pathway of Bcl-2 and reducing HDACi-induced histone H3 acetylation [160]. Highly elevated peroxisomes protect vorinostat-resistant lymphoma cells from ROS damage via two antioxidant mechanisms: (1) upregulating catalase and (2) increasing the levels of plasmalogens (PlsEtn) and related genes (such as GNPAT, FAR1 and FAR2) [161]. High levels of HSPA1A is associated with VPA resistance in lymphoid neoplasms. Inhibition of HSPA1A by KNK-437 could resensitize cells to VPA-induced apoptosis [162]. Furthermore, the phosphoproteins MAPKAPK2, ACTB, HSP90AA1 and HSP90AB1 were considered resistance hubs in VPA-resistant AML cell lines [163].

Altered levels of antiapoptotic proteins drive resistance against HDACi-mediated apoptosis. Specifically, it has been observed that increased JAK/STAT signaling negatively affects HDACi-induced death of CTCL cells and that high levels of phosphorylated STAT 3 is correlated with a lack of response to vorinostat [164]. Consistently, elevated levels of the antiapoptotic proteins BCL-2 and Bcl-xL show strong resistance to vorinostat-induced apoptosis in DLBCL cell lines [119]. Similarly, NF- κB upregulation confers Hodgkin’s lymphoma (HL) cell resistance to MGCD0103 and panobinostat by interfering with apoptosis [165]. Moreover, significant induction of NF-κB and its upstream regulator PD-L1 were found to be related to the resistance of myelodysplastic syndrome (MDS) and AML to LBH-589 therapy [166]. CDK inhibitors also act as key resistance-inducing factors. P21 and p27 have sustained overexpression in DLBCL, inhibiting cell cycle arrest and cell death induced by PXD101 [167]. Overexpression of p21 and p16 induces G1 arrest, increases SAHA- and depsipeptide-induced antiapoptotic genes and decreases proapoptotic genes in acute T cell leukemia cells [168]. Upregulation of p21 in acute promyelocytic leukemia (APL) cells attenuates HDACi-induced DNA damage and cell cycle arrest, which is correlated with DNA repair [167].

The generation of ROS is one of the key mechanisms by which HDACis induce cell death in malignant cells. However, the increased expression of vorinostat-induced antioxidant genes, such as glutathione (GSH), glutamate cysteine ligase (GCL) and superoxide dismutase (SOD), is related to HDACi resistance in advanced AML and MDS [169, 170]. In addition, properly activated autophagy promotes apoptosis in HDACi-treated cells. Conversely, a study showed that excessive activation of autophagy is necessary to protect the vorinostat-resistant lymphoma cell line and DLBCL cell line from apoptosis. HDAC6 deacetylation of HSP90 mediates chaperone complex assembly, and excessive autophagy to remove accumulated misfolded/aggregated proteins is considered a protective mechanism of autophagy against vorinostat-resistant cells [171]. To date, remarkably little is known about HDACi-induced multidrug resistance at single-cell levels. Elucidation of resistant mechanisms using a series of single-cell sequencing may contribute to overcome tumor heterogeneity for HDACi-resistance.

## Conclusion

As key deacetyltransferase subunits of multiprotein complexes, they regulate histone affinity for DNA and chromatin accessibility to their cognate binding proteins by compaction of DNA/histone complexes. Their biochemical and molecular characterization significantly affects the deacetyltransferase activity of HDAC-containing complexes. Importantly, the catalytic/noncatalytic and histone/nonhistone effects of HDACs on hematopoietic cells confer their ability to regulate a variety of cellular events in normal and malignant hematopoiesis. HDAC actions are gene or environment specific during hematopoiesis: (1) Different genes regulated by the same HDAC require the recruitment of different coregulators. For instance, HDAC1 has been found in at least three multiprotein complexes, including Sin3, CoREST and NuRD complexes. (2) One HDAC can act as a coactivator or corepressor on different genes and utilize different domains to act on interacting proteins. For instance, HDAC1-containing NuRD/MeCP1 corepressor complexes play an important role in GATA-1-mediated repression of target genes (i.e., GATA-2, γ-globin, c-myc, c-kit and Hes1), which are all required for the proliferation of hematopoietic progenitors. However, during GATA-1-mediated activation of the β-globin gene, the HDAC1/NuRD/MeCP1 complex is still recruited to the GATA-1 sites of the β-globin locus. (3) HDACs act as multifunctional regulators of transcription complex activity. For instance, HDAC1 can be acetylated by histone acetyltransferase p300. Acetylated HDAC1 not only loses its deacetylase activity but also inhibits the deacetylase activity of HDAC2, thereby downregulating the overall deacetylase activity of HDAC1/2-containing complexes, including the NuRD complex.

Epigenetic changes that occur during the development of hematopoietic malignancies are reversible and amenable to pharmacological intervention. The abnormal activity and expression of HDACs or the occurrence of aberrant composition in HDAC-containing transcriptional complexes could lead to malignant hematopoiesis via hyperproliferation and/or blocks in differentiation. However, the molecular basis for hematopoietic transformation, malignant development and drug resistance by HDACs are still largely unclear. For example, although it has been found that the activity of HDACs is regulated through posttranslational modifications, i.e., CBP/p300, and HDAC1 gene expression is regulated by the C/EBP family, GATA1 and Sp1, the misregulation in HDAC expression by multiple layers of regulation mechanisms, such as a given mutation or epigenetic modifier, are largely unknown. In some cases, the function of oncogenic TFs and fusion proteins is reliant on direct interactions with HDAC-containing complexes. For example, transcriptional and differential repression of several transcriptional fusion proteins with key roles in the progression of acute leukemias, such as AML1/ETO, STAT5/RARa, and PLZF/RARa fusion proteins, is mediated by the aberrant recruitment of corepressor complexes in the N-CoR/mSin3/HDAC1 complex. Hence, many HDAC–TF/cofactor interaction surfaces represent compelling therapeutic targets.

In hematopoietic systems, HDACis have shown synergistic or additive effects with numerous chemotherapeutic agents, such as proteasome inhibitors, hormonal therapy, tyrosine kinase inhibitors, DNA-hypomethylating agents, and immune checkpoint inhibitors, in preclinical and clinical settings. HDACis recently emerged as promising immunomodulatory drugs, like TMP195 and ACY241, via modulation of immune cell phenotypes or expression of immune checkpoints [80, 172]. Immune checkpoint inhibitors have revolutionized the treatment of hematological malignancies. Their combination with HDACis may be considered a major breakthrough in the treatment of hematological malignancies. Furthermore, more current research efforts are focused on developing HDAC isoform-selective inhibitors to improve toxicity against specific cancer types and overcome drug resistance or off-target effects. The availability of crystal structures of HDAC isoforms may provide a major contribution to understanding isoform selectivity.

Given that epigenetic regulation of HDACs globally affects the gene regulatory network, an ensemble of key hematopoietic HDACs has been identified. Future studies need to identify more regulatory factors that dysregulate HDAC expression and determine how their misexpression contributes to the pathogenesis of hematological malignancies. Identification of more structures of HDAC isoforms and epigenetic mechanisms, thereby targeting specific HDAC, HDAC pathways and HDAC-TF/cofactor interactions may represent ideal strategies to treat malignant hematopoiesis.

## Data Availability

Not applicable.

## Abbreviations

ALL: Acute lymphocytic leukemia
ALPs: All-lymphoid progenitors
AML MLL: Acute myeloid leukemia with a Mixed Lineage Leukemia
AML: Acute myeloid leukemia
APCs: Antigen-presenting cells
APL: Acute promyelocytic leukemia
BFU-E: Burst-forming unit-erythroid
BLPs: B cell-biased lymphoid progenitors
BMDMs: Bone marrow-derived monocytes
B-NHL: B cell non-Hodgkin lymphoma
CAR: Contractile actin ring
CFU-E: Colony-forming unit-erythroid
CK2: Casein kinase 2
cKO: Conditional knockout
CLL: Chronic lymphocytic leukemia
CLP: Common lymphoid progenitor
CML: Chronic myeloid leukemia
CMP: Common myeloid progenitor
CRc: Composite complete response
CTCL: Cutaneous T-cell lymphoma
DCs: Dendritic cells
DLBCL: Diffuse large B cell lymphoma
DN: Double-negative
DP: Double-positive
FL: Follicular lymphoma
GC: Germinal center
GCL: Glutamate cysteine ligase
GMCSFR: Granulocyte colony-stimulating factor
GMP: Granulocyte-monocyte progenitor
GSH: Glutathione
HAT: Histone acetyltransferase
HD: Hodgkin disease
HDACis: Histone deacetylase inhibitors
HDACs: Histone deacetylases
HL: Hodgkin’s lymphoma
HPCs: Hematopoietic progenitor cells
HSC: Hematopoietic stem cell
ISP: Immature single-positive
LMPP: Lymphoid-primed multipotential progenitor
MCSFR: Granulocyte-macrophage colony-stimulating factor
MDS: Myelodysplastic syndrome
MEP: Megakaryocyte- erythrocyte progenitor
MLP: Multilineage progenitor
MM: Multiple myeloma
NHL: Non-Hodgkin lymphoma
ORR: Overall response rate
PlsEtn: Plasmalogens
RORγt: Retinoic acid-related orphan receptor
RTEs: Recent thymic emigrants
SOD: Superoxide dismutase
TCR: T cell receptor
TF: Transcription factor
Tregs: T regulatory cells
