# Correction to: Role of HDACs in normal and malignant hematopoiesis

**DOI:** 10.1186/s12943-020-01182-w

**Published:** 2020-03-12

**Authors:** Pan Wang, Zi Wang, Jing Liu

**Affiliations:** 1 Hepatobiliary and Enteric Surgery Research Center, Xiangya Hospital, Central South University, Changsha, 410008 Hunan China; 2 Molecular Biology Research Center and Hunan Province Key Laboratory of Basic and Applied Hematology, School of Life Sciences, Central South University, Changsha, 410078 Hunan China

**Correction to: Mol Cancer**

**https://doi.org/10.1186/s12943-019-1127-7**

After the publication of this work [1], the authors note that there are no figure legends in the published paper. So we supplement the figure legends in this correction draft. The authors extend their apology for any inconvenience caused by this fault.

**The figure legends:**

**Fig 1. The classification of HDAC family.** Specific domains, subcellular localization and targeting inhibitors (HDACis) for the HDAC family. Some HDACis are HDAC isoform-selective inhibitors. RGFP966 is the selective inhibitor for HDAC3. Citarinostat and SLK-23bb are the selective inhibitors for HDAC6. Divalproex sodium is a selective inhibitor for SIRT1. Number 0 stands for the preclinical trial. Number 1, 2, 3 and 4 stands for phase I/II/III/IV clinical trials, respectively.

**Fig 2. Schematic representation of the main HDACs and HDAC-related TFs involved in hematopoietic lineage commitment.** Generally, HDACs (marked in red) regulate hematopoietic development by interplaying with specific TFs (marked in blue) at different stages. HDAC1/2 are widely involved in the development of hematopoietic lineages, while other HDACs, such as HDAC8, promote the differentiation of HSCs to progenitor cells. HDAC3 is mainly involved in the development of lymphoid lineages. HDAC5 mainly participates in the regulation of erythrocyte differentiation, while HDAC7, HDAC4 and HDAC11 are involved in the development of macrophages, dendritic cells and neutrophils, respectively.

**Fig 3. A model highlights component transformation in transcriptional complex is critical for leukemic transformation.** In normal myeloid differentiation, DNA-bound AML1 interacts with p300, MOZ, pCAF and nuclear receptor coactivators (CoA). This association results in an increase in histone acetylation, chromatin remodeling and transcriptional activation. While in t(8;21)AML, the AML1/ETO fusion recruits the NCoR/SMRT–Sin3a–HDAC complex, which leads to a histone acetylation reduction, chromatin organization inhibition and transcriptional repression, thus blocking myeloid differentiation.

**Fig 4. A model of CBP/P300 and HDAC component patterns determines the transcriptional function of TF in erythroleukemia cell differentiation.** HDAC1 interacts with the nuclear co-repressor mSin3A and associates with EKLF in undifferentiated EBHX11L cells. This co-repressor complex mediates β-globin gene transcriptional repression. During the differentiation of EBHX11L cells to a primitive erythroid phenotype, the formation of EKLF-P300/CBP-SWI/SNF complexes acetylates both EKLF and histones. P300/CBP-mediated EKLF acetylation decreases EKLF interaction with mSin3A-containing complexes. This leads to a conversion of EKLF from a transcriptional repressor to activator and promotes cellular differentiation.

**Fig 5. A model of class II HDAC interaction patterns in erythroid differentiation.** pERK phosphorylates the nuclear localization domain of HDAC5 and facilitates HDAC5-mediated shuttle of GATA1 and EKLF from the cytoplasm to the nucleus. Once the nuclear remodeling shuttle erythroid (NuRSERY) complex of pERK- HDAC5-GATA1-EKLF enters the nucleus, HDAC5 indirectly modulates the deacetylation of GATA1 and EKLF by recruiting HDAC3 to the complex. With erythroid maturation, HDAC5, GATA1 and EKLF remains associated but the levels of pERK sharply decreased, which leads to the shuttle of the complex from the cytoplasm to nucleus and dissociation of HDAC3.

**Fig 6. A model of TF modification affects the recruitment of HDAC to the promoter.** HDAC1 can be recruited by Ikaros to repress the demethylase of KDM58 in normal cells. In B-ALL, Casein kinase 2 (CK2)-mediated phosphorylation of Ikaros decreases HDAC1 recruitment to the KDM58 gene, which contributes to KDM58 expression and leukemogenesis.

**Fig 7. Abnormal gene expression of HDACs in human different hematological malignancies.** The most significantly upregulated HDAC genes are marked in red. The most prominently downregulated HDAC genes are marked in blue. CML: chronic myelocytic leukemia, AML: acute myelocytic leukemia, AML MLL: acute myelocytic leukemia with mixed lineage leukemia, CLL: chronic lymphocytic leukemia, ALL: acute lymphoblastic leukemia, MDS: myelodysplastic syndromes. The data are analyzed based on the TCGA database.

**Fig 8. Sensitivity and resistance mechanisms of hematological malignancies to HDACis.** The major anti-tumor actions of HDACi include HDACi-induced DNA damage, cell cycle arrest, differentiation, apoptosis, ROS and activated autophagy. The potential resistant mechanisms to HDACi include ABC transport-mediated drug efflux, increased DNA repair capacity, CDK/CDKI overexpression-induced normal cell cycle, strongly activated autophagy, multiple pathways-mediated apoptosis inhibition and PlsEtn overexpression-mediated inhibition of ROS damage.

**In order to improving literature evidence, additional five references are cited as supplementaries.**

1. Sun Y, Yang Y, Zeng S, Tan Y, Lu G, Lin G: **Identification of proteins related to epigenetic regulation in the malignant transformation of aberrant karyotypic human embryonic stem cells by quantitative proteomics.***PLoS One* 2014, **9:**e85823. (as a complement to reference1 and 2)

2. Han X, Zhang J, Peng Y, Peng M, Chen X, Chen H, Song J, Hu X, Ye M, Li J, et al: **Unexpected role for p19INK4d in posttranscriptional regulation of GATA1 and modulation of human terminal erythropoiesis.***Blood* 2017, **129:**226-237. (as a complement to reference 45)

3. Liang L, Peng Y, Zhang J, Zhang Y, Roy M, Han X, Xiao X, Sun S, Liu H, Nie L, et al: **Deubiquitylase USP7 regulates human terminal erythroid differentiation by stabilizing GATA1.***Haematologica* 2019, **104:**2178-2187. (as a complement to reference 45)

4. Sun X, Xiao Y, Zeng Z, Shi Y, Tang B, Long H, Kanekura T, Wang J, Wu H, Zhao M, et al: **All-Trans Retinoic Acid Induces CD4+CD25+FOXP3+ Regulatory T Cells by Increasing FOXP3 Demethylation in Systemic Sclerosis CD4+ T Cells.***J Immunol Res* 2018, **2018:**8658156. (as a complement to reference 59 and 60)

5. Zhou L, Li Y, Li X, Chen G, Liang H, Wu Y, Tong J, Ouyang W: **Propranolol Attenuates Surgical Stress-Induced Elevation of the Regulatory T Cell Response in Patients Undergoing Radical Mastectomy.***J Immunol* 2016, **196:**3460-3469. (as a complement to reference 61)

**The institution name: ‘1. The Xiangya Hospital, Central South University, Changsha 410005, Hunan, China’ change to the new one: ‘1. Hepatobiliary and Enteric Surgery Research Center, Xiangya Hospital, Central South University, Changsha 410008, Hunan, China’**
